# Evaluation of a 0.05 T low-field MRI system for canine brain imaging via comparison with images acquired at 1.5 T field strength: a pilot cadaver study

**DOI:** 10.1080/01652176.2026.2720640

**Published:** 2026-09-12

**Authors:** Elisabeth Maria Burgers, Samantha Miles, Stefanie Veraa, Thomas O'Reilly, Ruben van den Broek, Chloé Najac, Beatrice Lena, Andrew Webb

**Affiliations:** a Division of Diagnostic Imaging, Department of Clinical Sciences, Utrecht University, Utrecht, the Netherlands; b IDEXX Telemedicine Consultants, Department of Radiology, Hoofddorp, the Netherlands; c C.J. Gorter MRI Center, Department of Radiology, Leiden University Medical Center, Leiden, the Netherlands

**Keywords:** Canine neuroimaging, low field magnetic resonance imaging, image contrast, hydrocephalus, one health, sustainable imaging, permanent magnets, Halbach array

## Abstract

Magnetic resonance imaging (MRI) systems continue to grow in number in veterinary and human medicine with higher field strength magnets increasingly available; however, the associated high costs have created healthcare inequities. This technical feasibility study aimed to evaluate a low-cost, portable, and permanent magnet-based 0.05 T low-field system (LF), originally designed for human neuroimaging, for assessing the canine brain. By using 23 canine cadavers, a scanning protocol was developed, consisting of transverse T1-weighted (T1w) and T2-weighted (T2w) sequences, and compared to a 1.5 T (HF) protocol for the visibility of 22 anatomical features. For the purpose of hydrocephalus evaluation, the LF system showed non-inferior detection of the lateral ventricles (LV) and mesencephalic aqueduct on T2w sequences with >90% conditional probability for detecting these features on the LF, given they were present on the HF. For T1w sequences, the LF system had inconclusive non-inferiority for detection of LV with 76.6% conditional probability. In summary, a canine brain scanning protocol was successfully developed for a 0.05 T MRI with preliminary evaluation suggestive that this device can aid with the detection of hydrocephalus. Future *in vivo* studies are required to evaluate the device's true clinical application.

## 1. Introduction

Clinical usage of magnetic resonance imaging (MRI) continues to grow in both veterinary and human medicine with an increasing number of MRI scanners available in both veterinary schools and private practice. (Kang et al. 2009; Gavin 2011; Hespel and Cole 2018; Jacqmot et al. 2022; Veterinary MRI Market - By Solution MRI Scanner (2023) The higher soft-tissue contrast of MRI compared to computed tomography (CT) means that it is indispensable as a neuroimaging modality, particularly for evaluation of abnormalities related to the brain and spinal cord. (Kang et al. 2009; Hespel and Cole 2018; Jacqmot et al. 2022).

MRI scanners are classified according to their magnetic field strength, expressed in millitesla (mT) or Tesla (T), and are broadly categorized as ultra-low-field (<10 mT), low-field (10–200 mT), mid-field (200 mT−1.5 T), high-field (1.5–3 T), and ultra-high-field (>3 T). (Hespel and Cole 2018; Geethanath and Vaughan 2019; Jacqmot et al. 2022). Over a decade ago, mid-field MRI was reported to predominate in veterinary practice, however higher field strengths are increasingly available with various veterinary studies in the last decade evaluating 3 T and 7 T imaging. (Kang et al. 2009; Bolen et al. 2010; Konar and Lang 2011; Martin-Vaquero et al. 2011; Datta et al. 2012; Anaya García et al. 2015; Jacqmot et al. 2017; Hespel and Cole 2018; Czeibert et al. 2019; Nitzsche et al. 2019; Johnson et al. 2020; Jacqmot et al. 2022). Spatial resolution influences MRI image quality and is intrinsically linked to the signal-to-noise ratio (SNR), which increases with higher field strengths. This increased SNR can be leveraged for a finer spatial resolution to improve lesion localization and characterization. The broader diagnostic capabilities of higher field strength magnets have helped drive the development of MRI, particularly for general clinical usage. (Kang et al. 2009; Hespel and Cole 2018; Geethanath and Vaughan 2019; Marques et al. 2019; Sarracanie and Salameh 2020; Wald et al. 2020; Jacqmot et al. 2022). However, high-field systems are expensive with average purchasing costs of approximately $1 million per Tesla that are compounded by additional fees, such as installation and maintenance contracts. (Konar and Lang 2011; Hespel and Cole 2018; Geethanath and Vaughan 2019; Marques et al. 2019; O'Reilly et al. 2019; Sarracanie and Salameh 2020; Wald et al. 2020) Due to these prohibitive costs, resource-limited areas have reduced access to MRI with consequential global healthcare inequities.

In terms of human healthcare, this inequity has renewed interest in the development of low-field MRI to reduce the cost and required infrastructure usually associated with high-field systems, thereby improving global accessibility. Various technological advancements have enabled the acquisition of diagnostically useful information from the lower SNR inherent to low-field strengths, such as improved permanent magnet designs and image processing algorithms. (Geethanath and Vaughan 2019; Marques et al. 2019; O'Reilly et al. 2019; Sarracanie and Salameh 2020; Wald et al. 2020; O'Reilly et al. 2021; de Vos et al. 2021; Obungoloch et al. 2023). The clinical value of a low-cost, low-field MRI can be further improved by features such as portability and a focused point-of-care applicability. (O'Reilly et al. 2019; Sarracanie and Salameh 2020; Wald et al. 2020; 2021) With these goals, O'Reilly et al. (2019) designed a low-cost, portable, low-field MRI system for neuroimaging of human pediatric hydrocephalus in resource-limited locations. (O'Reilly et al. 2019) This device consists of a 27-cm clear-bore Halbach array of permanent magnets that produces a main magnetic field strength (B_0_) of approximately 50 mT (0.05 T) with additional shimming rings to compensate for material and construction imperfections. Early *in vivo* studies with this system show that images of the human brain can be acquired with a reasonable data-acquisition time. (O'Reilly et al. 2019; O'Reilly et al. 2021; de Vos et al. 2021; Obungoloch et al. 2023). Current Halbach-based systems have larger diameters, typically 30–32 cm, and the original system became available for this study.

The first objective of this study was to scan a diverse population of canine cadavers to develop a canine brain protocol using the original system designed by O'Reilly et al. (2019). The second objective was an exploratory clinical assessment via the comparative identification of various anatomic features between 0.05 T (LF) and 1.5 T (HF) acquired images. As canine patients frequently have variable degrees of ventriculomegaly, this may form an interesting animal model for the initially intended purpose of the device for human pediatric hydrocephalus neuroimaging. (Wisner and Zwingenberger 2015; Mai 2018; de Lahunta et al. 2021).

## 2. Materials and methods

### 2.1. Cadaver selection

As this was a post-mortem study, no ethical committee approval was required. All cadavers were acquired via an informed consent donation program at the primary author's institution. When available, information regarding signalment, clinical history, and cause of death was recorded. All cadavers were less than 30-kg, per the scheme's guidelines for donation, and were included if the head was expected to fit within the radiofrequency coils of the LF system.

### 2.2. Low-field MRI

The LF system is the original unit developed by O'Reilly et al. and is summarized in previous works. (O'Reilly et al. 2019; O'Reilly et al. 2021; de Vos et al. 2021; Obungoloch et al. 2023). The magnet has a length of approximately 50 cm, diameter of 27 cm, and a magnetic field strength (B_0_) of approximately 50 mT (0.05 T). The bore is open at both ends to facilitate cadaver positioning. All imaging was performed with solenoidal coils with diameters of 23 cm and 28 cm, for smaller or larger dogs.

### 2.3. Data collection

Cadavers were positioned in sternal recumbency for all scans with the selected coil and in-plane field-of-view (FOV) dependent on head size.

Images were acquired on the LF system as 3D data sets, comprising a transverse T1-weighted (T1w) turbo spin-echo (TSE) and either a T2-weighted (T2w) and/or Driven Equilibrium Fourier Transform (DEFT) sequence. The DEFT sequence was utilized to produce images comparable to a T2w sequence with improved signal for comparable or reduced acquisition time. Due to the developmental nature of the study, a T2w or DEFT sequence was not available in all cadavers. After acquisition, the images were reconstructed with a standard Fast Fourier Transform and an intensity correction for the RF coil profile was applied. (Lena et al. 2025).

The HF system (Ingenia, Philips Healthcare, Eindhoven, the Netherlands) at the primary author's institution was used to acquire transverse T1w 2D multi-slice SE and T2w TSE sequences using either a dedicated 20-channel head and neck coil or an 8-channel small extremity coil, depending on cadaver size. Image reconstruction and processing followed the standard Philips (vendor-protected) reconstruction pipeline.

### 2.4. Image assessment

Based on the findings of a previous study by Jacqmot et al. (2022), a total of 22 anatomical features were selected: regional anatomy distinction (Reg), cerebral gyri/sulci distinction (GS-C), cerebellar gyri/sulci distinction (GS-Cb), cerebral grey/white-matter distinction (GWM-C), lateral ventricles (LV), septum pellucidum (SP), third ventricle (TV), mesencephalic aqueduct (MA), fourth ventricle (FV), optic pathway (CN−2), falx cerebri (Falx), internal capsule (IntCap), corpus callosum (CC), sella turcica (ST), caudate nuclei (Caud), thalamus (Thal), interthalamic adhesion (ITA), rostral/caudal colliculi (Col), lingula/nodulus (LingNod), vermis, flocculonodular lobe (Floc), cochlea/internal acoustic meatus (IAM). (Jacqmot et al. 2022). To account for slightly different slice locations between 3D LF and 2D HF scans, as well as through slice partial volume effects, the definitions of a few anatomic features were broadened to include identification of any number of defined parts. For example, CN−2 was defined as the ability to identify either the optic nerve just prior to the canal, in the optic canal, and/or the optic chiasm. This method was also utilized for the Col, LingNod, and IAM. Feature identification was aided by various anatomical and MRI references and scored via a binary visibility rating scale defined as follows: 1 = clearly defined and/or unambiguously visible or 0 = ill-defined, poorly differentiated, or not visible. (Kang et al. 2009; Martín-Vaquero et al. 2011; Wisner and Zwingenberger 2015; Mai 2018; Czeibert et al. 2019; Hermanson and de Lahunta 2019; de Lahunta et al. 2021; Jacqmot et al. 2022).

All sequences from each cadaver were classified by system and protocol into four groups, (1) LF T1w, (2) LF T2w or DEFT, (3) HF T1w, and (4) HF T2w, and assigned a randomized numerical identifier within each scoring session (https://www.random.org/lists/). The order of evaluation per scoring session was HF T1w, LF T1w, HF T2w, and LF T2w or DEFT, with a minimum of one week delay between assessment of the HF and LF studies. All included sequences were retrospectively and independently scored twice, at least four weeks apart, by two observers, E.M. (2nd year veterinary radiology resident of the European College of Veterinary Diagnostic Imaging, ECVDI) and S.M. (Board-certified radiologist of the American College of Veterinary Radiology). All images were evaluated in DICOM format on a radiological workstation with medical-grade monitors using dedicated software (RadiAnt DICOM Viewer, Medixant, Poznań, Poland; Enterprise Imaging Platform, AGFA HealthCare, Mortsel, Belgium).

### 2.5. Statistical analysis

Statistical analysis was performed using commercially available software (Microsoft Excel version 2502, Microsoft; Python version 3.10, Python Software Foundation). Unless otherwise specified, all analyses were done per system-sequence group and scoring session. If applicable, a *p*-value < 0.05 was considered statistically significant.

A Gwet's AC1 (*κ*) was used to assess inter- and intra-observer reliability due to its improved reliability in situations of high agreement or skewed scoring distributions. (Gwet 2008) Results are reported with a 95% confidence interval (CI) and probabilistic benchmark as per Landis and Koch: <0.00, poor; 0.00–0.20, slight; 0.21–0.40, fair; 0.41–0.60, moderate; 0.61–0.80, substantial; 0.81–1.00, almost perfect agreement. (Landis and Koch 1977) Anatomic features were included for further analysis if the interobserver reliability was at least moderate (κ ≥ 0.41) for both systems in both scoring sessions.

For each included feature, a 2 × 2 contingency table was created and the visibility rates per system, defined as the proportion of ‘1’ scores, was calculated. The difference in visibility rates between the systems, Δ(LF–HF), is reported with a 95% CI. The systems were compared using a two-tailed McNemar's test with either chi-square approximation (≥25 discordant pairs) or an exact test (<25 discordant pairs). Non-inferiority of the LF system was assessed using a 15% margin, defined *a priori* as the maximum acceptable Δ(LF–HF). A more liberal margin was chosen due to the exploratory nature of the study. If the lower bound CI of Δ(LF–HF) was greater than −15% with CI width <20%, the LF system was considered non-inferior. Non-inferiority assessment was inconclusive if the McNemar *p*-value was > 0.05 and the CI width too wide (>20%) for precise point estimation. To complement these results, the conditional probability for a feature to be present on the LF system, if identified on the HF, was calculated (P[LF+|HF+]).

Descriptive statistics were used to provide a cadaver-level overview of the number of features detected per cadaver. A feature was considered detected if it was visible in at least two of four observations. The number of features present per cadaver between the two systems was compared using a one-sided Wilcoxon signed-rank test for each sequence type.

## 3. Results

### 3.1. Scan data

Twenty-three canine cadavers were included with a mean weight of 14.2 kg (*n* = 4, range 5.6–22.8 kg). There were 17/23 (74%) dogs with mesaticephalic and 6/23 (26%) with brachycephalic conformations. The majority of cadavers were donated from external clinics, therefore signalment, clinical history, and cause of death were frequently unknown (Supplementary Table 1).

All scanning for a cadaver was performed on the same day. Table 1 provides an overview of the most commonly selected acquisition parameters with the average acquisition time for each sequence per system. Individual acquisition parameters per cadaver are available in Supplementary Table 2. When more than one NSA was acquired, the images were averaged.

**Table 1.** Representative acquisition parameters for the transverse sequences per system. The mode of each parameter is presented, except for acquisition time which is shown as the mean. There are 23 cadavers for each sequence unless otherwise specified. Acquisition information per cadaver is available in Supplementary Table 2.

	0.05 T			1.5 T	
	T1w	T2w (*n* = 20)	DEFT (*n* = 7)	T1w	T2w
TR (ms)	600	1500	400	694	6014
TE (ms)	13	13	50	14	100
Echo train length (ETL)	10	15	4	1	16
In-plane FOV (mm)	120 × 120	120 × 120	150 × 150	119 × 119	120 × 120
Slice thickness (mm)	3	3	3	3	3
Number of averages	2	2	1	2	4
Acquisition time (min)	05:16	09:16	10:11	04:47	05:01

The LF studies were acquired either off-site at the developing institution (14/23; Leiden University Medical Center) or at the primary author's institution after temporary device placement (9/23; Utrecht University). Available sequences included transverse T1w in 23/23 (100%), transverse T2w in 20/23 (87%), and DEFT in 7/23 (30%). One cadaver (4%) had neither a T2w or DEFT sequence. Five cadavers (5/23, 22%) had both a T2w and DEFT sequence of which only one was included for the purposes of further image evaluation. This was the T2w in 2/5 (cadavers #19, #21) and the DEFT in 3/5 (cadavers #15, #17, #21). The decision for inclusion was based on observer consensus and related to previous clinical experience assessing studies for diagnostic quality, namely related to the degree of noise and grey/white-matter distinction. For simplification in the following sections, ‘LF T2w’ refers to either the LF T2w or DEFT acquired sequences, unless otherwise specified.

All cadavers (23/23, 100%) had HF transverse T1w and T2w sequences.

### 3.2. Image analysis

#### 3.2.1. Inter- and intra-observer agreement

In the T1w sequences, 10/22 features were excluded from further analysis based on the interobserver agreement for both systems in both scoring sessions (Supplementary Table 3). For the 12/22 included features, the lowest observed interobserver probabilistic benchmark was moderate in 7/12 (κ_GS-C_ 0.56, κ_LV_ 0.43, κ_TV_ 0.46, κ_MA_ 0.58, κ_FV_ 0.56, κ_ST_ 0.59, κ_Col_ 0.48), substantial in 3/12 (κ_GWM-C_ 0.79, κ_Falx_ 0.60, κ_Caud_ 0.79), and almost perfect in 2/12 (κ_GS-Cb_ 0.85, κ_IntCap_ 0.85). Of the included features, the majority had at least substantial intraobserver agreement for each observer in the LF (E.B. 11/12, S.M. 10/12) and HF systems (E.B. 11/12, S.M. 9/12).

In T2w sequences, 7/22 features were excluded from further analysis based on the interobserver agreement for both systems in both scoring sessions (Supplementary Table 4). For the 15/22 included features, the lowest observed interobserver probabilistic benchmark was moderate in 4/15 (κ_FV_ 0.42, κ_Falx_ 0.44, κ_ST_ 0.44, κ_Vermis_ 0.52), substantial in 4/15 (κ_GS-C_ 0.66, κ_SP_ 0.69, κ_TV_ 0.8, κ_MA_ 0.69), and almost perfect in 7/15 (κ_Reg_ 0.95, κ_GWM-C_ 0.81, κ_LV_ 1.00, κ_IntCap_ 0.88, κ_CC_ 0.88, κ_Caud_ 0.94, κ_Thal_ 0.84). Of the included features, the majority had almost perfect intraobserver agreement for each observer in the LF (E.B. 10/15, S.M. 11/15) and HF systems (E.B. 15/15, S.M. 14/15).

#### 3.2.2. Feature visibility

Table 2 shows the results for system comparisons for T1w sequences. Feature visibility ranged from 1.1% (GS-Cb) to 93.5% (GWM-C) in the LF and 12.0% (GS-Cb) to 98.9% (Falx, ST) in the HF. Statistically non-significant system differences (*p*-value > 0.05) were identified for 6/12 features (GS-C, GWM-C, LV, TV, IntCap, Caud). Of these, the non-inferiority of the LF system was identified for 3/6 features (GWM-C, IntCap, Caud) with inconclusive non-inferiority of the remainder due to wide CI (GS-C, LV, TV). For all included features, the mean P(LF+|HF+) was 50.1% (SD 38.9%, range 0%–96.5%). For the 3/12 non-inferior features, the P(LF+|HF+) improved with a mean of 95.6% (SD 1.31%).

**Table 2.** System comparisons for T1w sequences per feature (*N* = 92 scores) with McNemar testing and non-inferiority analysis. Unless otherwise specified, all calculated *p*-values are from a McNemar exact test. Results are complemented with P(LF+|HF+), assessing the probability that the LF system detects a feature given that it is detected with the HF.

Feature	LF visibility *n* (%)	HF visibility​​​​​​ *n* (%)	Δ (LF–HF) % (95% CI)	*p*-value	Non-inferiority (15% margin)	P(LF+|HF+)
GS-C	9 (9.8%)	19 (20.7%)	−10.9 (−21.7, 0.0)	0.078^a^	Inconclusive	5.3%
GS-Cb	1 (1.1%)	11 (12.0%)	−10.9 (−18.2, −3.5)	0.006	No	0.0%
GWM-C	86 (93.5%)	81 (88.0%)	5.4 (−1.6, 12.5)	0.227	Yes	96.3%
LV	67 (72.8%)	77 (83.7%)	−10.9 (−21.7, 0.0)	0.078^a^	Inconclusive	76.6%
TV	8 (8.7%)	25 (27.2%)	−18.5 (−29.1, −7.8)	1.000^a^	Inconclusive	16.0%
MA	8 (8.7%)	24 (26.1%)	−17.4 (−27.4, −7.4)	0.001*	No	20.8%
FV	7 (7.6%)	17 (18.5%)	−10.9 (−19.4, −2.3)	0.021*	No	23.5%
Falx	76 (82.6%)	91 (98.9%)	−16.3 (−25.1, −7.5)	<0.001*	No	82.4%
IntCap	89 (96.7%)	85 (92.4%)	4.3 (−2.4, 11.1)	0.344	Yes	96.5%
ST	70 (76.1%)	91 (98.9%)	−22.8 (−33.0, −12.6)	<0.001*	No	75.8%
Caud	85 (92.4%)	85 (92.4%)	0.0 (−6.7, 6.7)	1.000	Yes	94.1%
Col	15 (16.3%)	38 (41.3%)	−25.0 (−37.6, −12.4)	<0.001^a^ ^,^ *	No	23.7%

^*^ *p*-value < 0.05.

^a^ Asymptotic *p*-value.

Table 3 shows the results for system comparisons for T2w sequences. Feature visibility ranged from 61.4% (FV) to 100.0% (LV) in the LF and 90.9% (FV) to 100.0% (Reg, GS-C, GWM-C, LV, Falx, IntCap, CC, ST, Caud, Thal) in the HF. Statistically non-significant differences (*p*-value > 0.05) were identified for only 2/12 features (LV, MA) with both features showing non-inferiority of the LF system. Non-inferiority of the LF system was identified with 3/12 additional features (Reg, SP, Thal) despite statistically significant differences between the systems (*p*-value < 0.05). Compared to T1w sequences, the T2w sequences showed a relatively higher P(LF+|HF+) for all included features with a mean of 85.5% (SD 8.22%, range 65%–100%). For the 5/12 non-inferior features, the mean P(LF+|HF+) was 93.0% (SD 4.0%).

**Table 3.** System comparisons for T2w sequences per feature (*N* = 88 scores) with McNemar testing and non-inferiority analysis. Unless otherwise specified, all calculated *p*-values are from a McNemar exact test. Results are complemented with P(LF+|HF+), assessing the probability that the LF system detects a feature given that it is detected with the HF.

Feature	LF visibility *n* (%)	HF visibility *n* (%)	Δ (LF–HF) % (95% CI)	*p*-value	Non-inferiority (15% margin)	P(LF+|HF+)
Reg	81 (92.0%)	88 (100.0%)	−8.0 (−13.6, −2.3)	0.016*	Yes	92.0%
GS-C	71 (80.7%)	88 (100.0%)	−19.3 (−28.1, −10.5)	<0.001*	No	80.7%
GWM-C	77 (87.5%)	88 (100.0%)	−12.5 (−19.6, −5.4)	0.001*	No	87.5%
LV	88 (100.0%)	88 (100.0%)	0.0 (0.0, 0.0)	1.000	Yes	100.0%
SP	80 (90.9%)	87 (98.9%)	−8.0 (−14.3, −1.6)	0.039*	Yes	90.8%
TV	72 (81.8%)	85 (96.6%)	−14.8 (−23.6, −6.0)	0.002*	No	82.4%
MA	75 (85.2%)	81 (92.0%)	−6.8 (−13.6, −0.1)	0.109	Yes	90.1%
FV	54 (61.4%)	80 (90.9%)	−29.5 (−41.2, −17.9)	<0.001^a^ ^,^ *	No	65.0%
Falx	76 (86.4%)	88 (100.0%)	−13.6 (−21.0, −6.3)	<0.001*	No	86.4%
IntCap	78 (88.6%)	88 (100.0%)	−11.4 (−18.1, −4.6)	0.002*	No	88.6%
CC	76 (86.4%)	88 (100.0%)	−13.6 (−21.0, −6.3)	<0.001*	No	86.4%
ST	72 (81.8%)	88 (100.0%)	−18.2 (−26.7, −9.7)	<0.001*	No	81.8%
Caud	74 (84.1%)	88 (100.0%)	−15.9 (−23.9, −7.9)	<0.001*	No	84.1%
Thal	81 (92.0%)	88 (100.0%)	−8.0 (−13.6, −2.3)	0.016*	Yes	92.0%
Vermis	65 (73.9%)	87 (98.9%)	−25.0 (−35.0, −15.0)	<0.001*	No	74.7%

^*^ *p*-value < 0.05.

^a^ Asymptotic *p*-value.

#### 3.2.3. Detected features per cadaver

**Figure 1.**
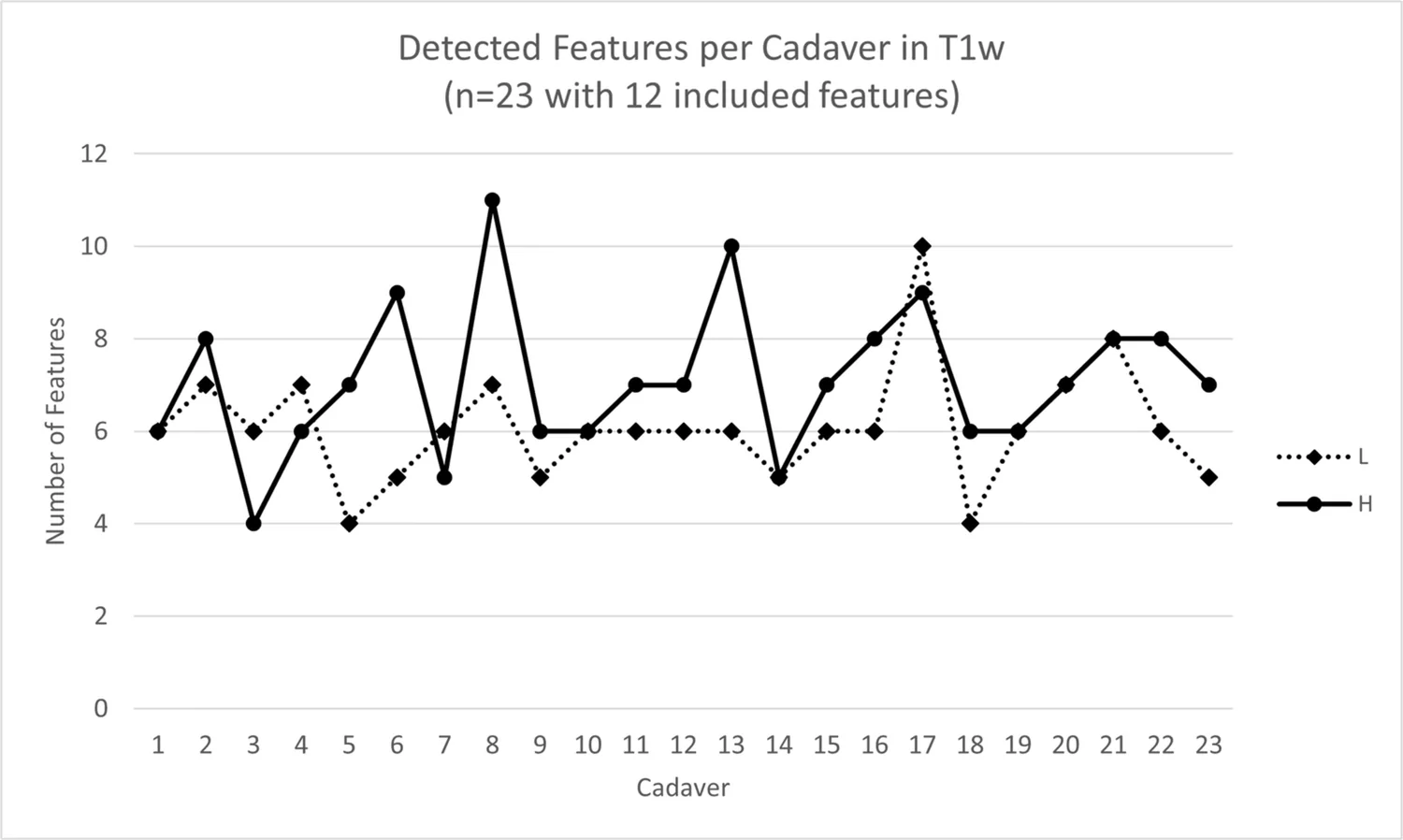
Paired-line graph indicating the number of detected features in T1w sequences per cadaver with each system. Features were considered detected if visible in at least two of four observations.

In T1w sequences, the mean detected features per cadaver for the LF was 6.1 (SD 1.3, range 4–10) and for the HF 7.1 (SD 1.6, range 4–11). The number of features detected per cadaver for each system is shown as a paired line-graph in Figure 1. The T1w sequence had relatively lower and inconsistent feature detection in both systems when compared to T2w.

**Figure 2.**
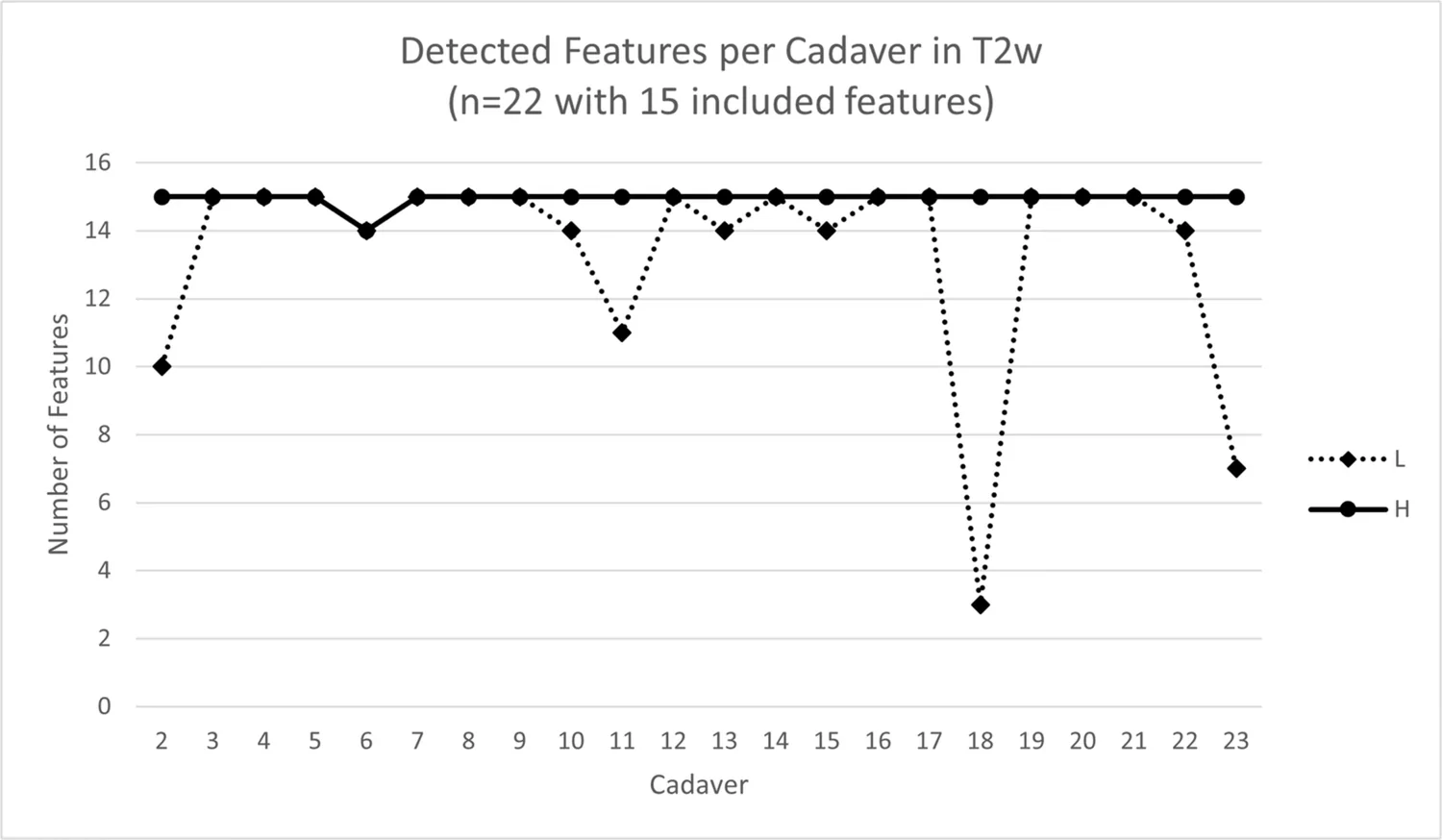
Paired-line graph indicating the number of detected features in T2w sequences per cadaver with each system. Features were considered detected if visible in at least two of four observations.

In T2w sequences, the mean detected features per cadaver for the LF was 13.5 (SD 3.1, range 3–15) and for the HF was 15.0 (SD 0.2, range 14–15). Figure 2 showing the number of features detected per cadaver for each system shows few outliers with the LF that had a low number of detected features (cadavers #2, 10, 18, 23). When excluding these, the mean detected features per cadaver with the LF system became more consistent and improved to 14.6 (SD 1.0, range 11–15).

The one-sided Wilcoxon signed-rank tests showed that overall visibility scores per cadaver were significantly higher in the HF relative to the LF for both T1w (W+ = 126.5, *n* = 17, *p*-value < 0.05) and T2w (W+ = 36, *n* = 8, *p*-value < 0.05) sequences.

## 4. Discussion

In this technical feasibility study, a diverse population of canine cadavers was used to develop a canine brain scanning protocol for a LF MRI (0.05 T). Comparison of the LF acquired images with those from a HF MRI showed non-inferiority of the LF system, with an exploratory 15% margin, for the identification of multiple anatomic features, particularly in the T2w sequences. Across both sequences, identification of the LV was the only overlapping feature with clear non-inferiority on T2w with 100% P(LF+|HF+) and inconclusive non-inferiority on T1w with 76.6% P(LF+|HF+). This is a relevant finding for the device's intended purpose for pediatric hydrocephalus neuroimaging, which is further supported by non-inferior detection of the MA on T2w with 90.1% P(LF+|HF+). The exclusion of multiple variables in both T1w and T2w sequences due to inconsistent interobserver agreement reflects the limitations of the current study, described in detail in the subsequent paragraphs.

As routine brain protocols in veterinary medicine commonly utilize T1w and T2w sequences, both were evaluated in this study. (Wisner and Zwingenberger 2015; Mai 2018) Excluding the few outliers, the LF T2w sequences had subjectively improved contrast resolution and more consistent feature identification relative to the T1w sequences. However, T2w sequences generally have lower SNR and require longer acquisition times to achieve an acceptable SNR. In a veterinary context, the small size of canine patients, particularly toy breeds commonly affected by hydrocephalus, presents an additional challenge for obtaining sufficient SNR. (Mai 2018) As T1 relaxation time decreases with decreasing B_0_, the major advantage of the LF T1w sequence is that it can achieve relatively high signal in a short acquisition time. (Marques et al. 2019; O'Reilly et al. 2021; Hori et al. 2021) In the current study, T1w sequences had a subjectively better SNR with shorter acquisition time (mean 05:16 min, range 02:08–10:06 min) relative to T2w (mean 09:16 min, range 04:43–27:00 min) or DEFT (mean 10:11 min, range 06:17–13:44 min). Future sequence development, particularly for veterinary medicine, should prioritize short acquisition times with higher SNR.

**Figure 3.**
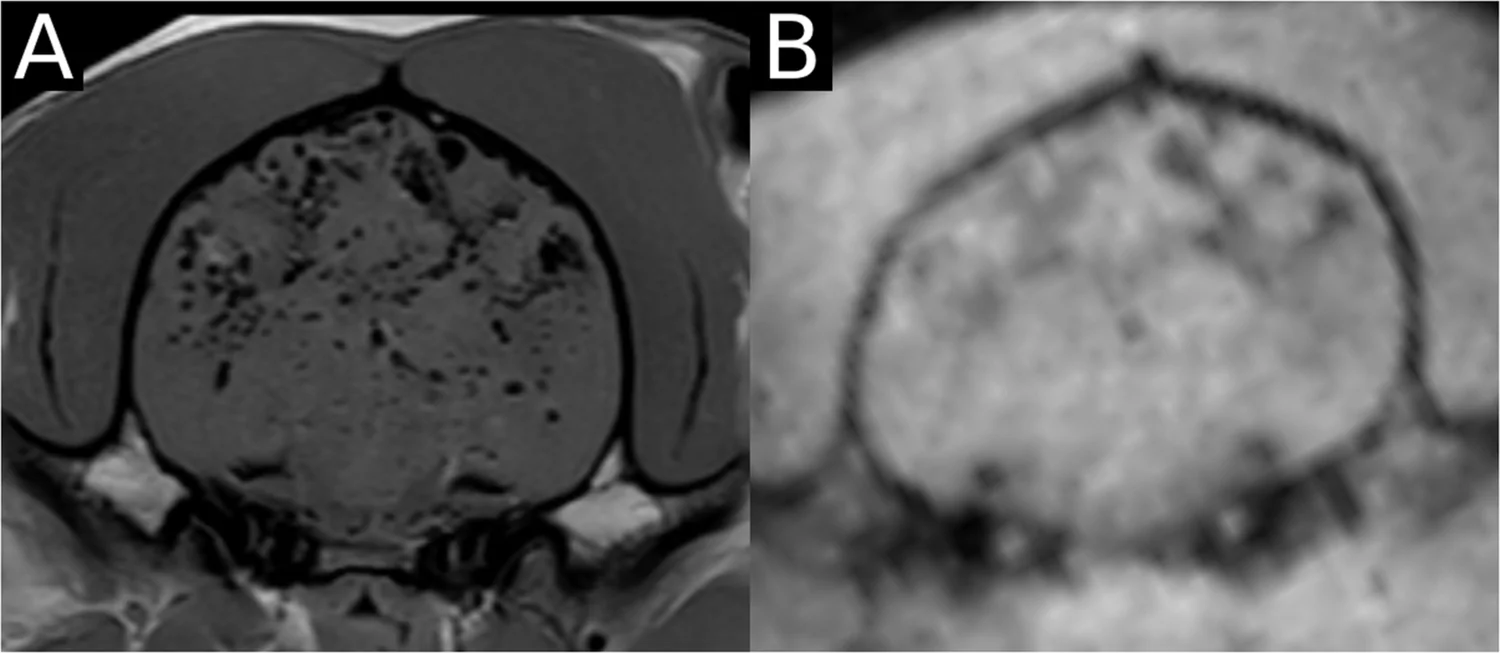
Transverse T1w images from cadaver #7 at the level of the midbrain with a large number of signal voids on the (a) HF acquired images. Visibility of these signal voids is markedly reduced on the corresponding (b) LF image compatible with reduced magnetic susceptibility. Dorsal is to the top and right to left of the images.

Post-mortem artifacts were a major limiting factor affecting all sequences with 22/23 cadavers having been frozen and thawed prior to imaging. The majority of artifacts were inherent to progressive tissue degradation and dehydration which results in variable fluid and biochemical composition of the cells, collapse of cerebrospinal fluid spaces, and focal regions of signal loss. For the current study, multiple cadavers had an absent to ill-defined gray-white matter distinction, particularly in T1w sequences, that limited delineation of various structures. This loss of distinction occurs due to progressive demyelination post-mortem and T1w sequences are more sensitive to this issue. (Schmitz et al. 2005) Additionally, freezing of the cadavers exacerbates tissue degradation through direct damage via cell lysis or formation of ice crystals that disrupt the tissue matrices. (Longson et al. 1995; Corfield et al. 2008; Kurmis et al. 2009; Kobayashi et al. 2010) Subsequent thawing, if done too quickly, results in further autolysis that influences tissue signal. These focal regions of signal loss secondary to degradation present as signal voids that can limit identification of normal anatomic structures. (Corfield et al. 2008; Kurmis et al. 2009) As higher strength magnets have increased sensitivity for small inhomogeneities of B_0_, in the current study these signal voids represented a larger problem in the HF images and were often negligible in the LF (Figure 3). (Konar and Lang 2011; Hori et al. 2021; Jacqmot et al. 2022). Furthermore, low cadaver temperatures likely contributed to altered tissue contrast and reduced T1 and, particularly, T2 signal, as reported in previous studies. (Tofts et al. 2008; Kurmis et al. 2009; Bolen et al. 2010; Kobayashi et al. 2010; Murray and Werpy 2010; Ruder et al. 2012; Zech et al. 2015) As all cadavers were thawed at room temperature, an inherent variability could be present dependent on the ambient environment. The reduced signal secondary to low temperature presented a greater challenge in the LF scans due to an inherently lower SNR compared to the HF, and particularly for the T2w sequences as discussed above. (Kang et al. 2009; Hespel and Cole 2018; Geethanath and Vaughan 2019; Marques et al. 2019; Sarracanie and Salameh 2020; Wald et al. 2020; Jacqmot et al. 2022). Various adjustments of sequence parameters, such as number of averages, allowed for partial recuperation of SNR, though this could be at the detriment of acquisition time. While long acquisition times were inconsequential in the current study, this method cannot be utilized in future *in vivo* studies with anesthetized canine patients. While the utilization of cadavers was a necessary first step for assessing the feasibility of the LF system for canine brain imaging, further development will require *in vivo* evaluation to overcome the multitude of post-mortem artifacts.

It was elected to use a binary rating scale for feature identification due to the exploratory and post-mortem nature of the current study. However, the initial T1w scoring session had multiple features with poor to slight interobserver agreement (κ ≤ 0.20), particularly in the LF system (Reg, SP, CN-2, Vermis, IAM), concerning for inconsistent implementation of the scoring protocol between observers. Prior to the second scoring session, an additional training with the T1w sequences was implemented to re-define these problematic features. While this improved the interobserver agreement for CN-2, CC, and ITA to at least substantial (κ ≥ 0.61) in both systems, these features were not included for further analyses due to the protocol change. Concerns for inconsistent protocol implementation were less evident in the initial T2w scoring. In human medicine, there are well-defined criteria for anatomic landmarks that can be used to assess new techniques or devices with imaging criteria or visual characteristic grading analysis studies. (Ludewig et al. 2010; Precht et al. 2019) However, these criteria are lacking in veterinary medicine, which limits the extent of consistent image evaluation. Furthermore, consistent image evaluation will be further hampered by inherent differences in sequence tissue weighting and, relevant to this study, the aforementioned post-mortem artifacts. Future studies can be strengthened by *in vivo* assessment that implements improved imaging criteria, additional observer training, and increased sample size and number of observers that, for example, would allow for visual grading characteristic analysis. (Månsson 2000; Båth and Månsson 2007; Ludewig et al. 2010; Tesselaar et al. 2016; Precht et al. 2019; Båth) Additionally, a broader scoring scale, such as 1–3, would allow for weighted agreement analysis.

**Figure 4.**
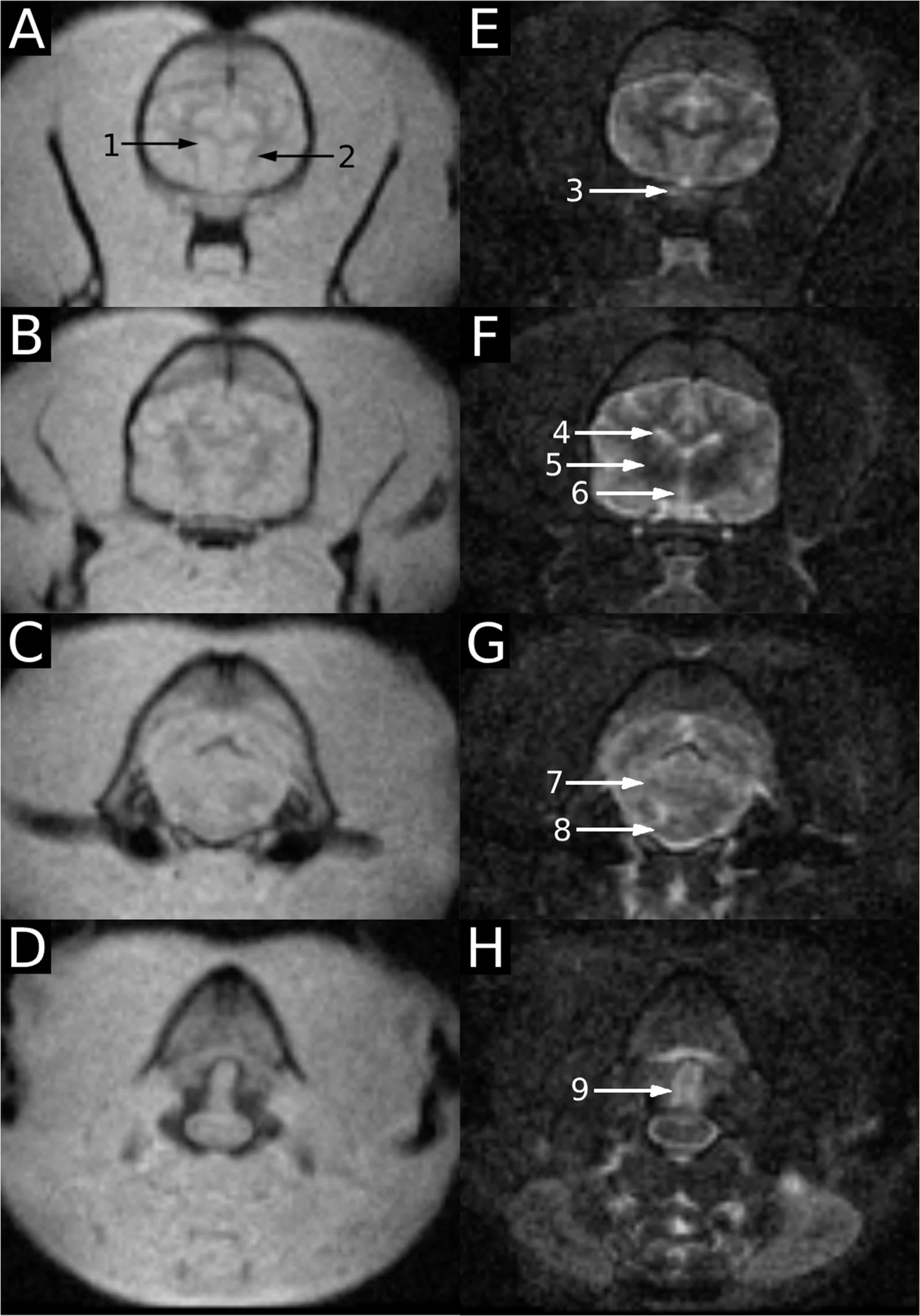
Transverse LF acquired (a–d) T1w and (e–h) T2w images from cadaver #14, which had clear distinction of the regional anatomy and cerebral grey-white matter in both sequences. Images are at the level of the (a, e) caudate nuclei (1), (b, f) thalamus (5), (c, g) rostral cerebellum (7) and brainstem (8), and (d, h) cerebellar vermis (9) and foramen magnum. The T2w images had subjectively lower SNR relative to the T1w. Dorsal is to the top and right to left of the images. Some of the assessed features are labelled: 1 = Caud, 2 = IntCap, 3 = CN-2 (optic nerve within canal), 4 = LV, 5 = Thal, 6 = TV, 7 = Cerebellum, 8 = Brainstem, 9 = Vermis.

For optimal scanning, certain changes should be implemented to the hardware and data acquisition protocol. The system is originally designed for human neuroimaging, where the size and geometry of the head varies very little between individuals of a certain age. The RF coils were designed with this in mind and tailored for the elliptical-dome shape of the human head with the accompanying geometry of a relatively thin, long neck. The shape of the canine head is much different, with the brain occupying a smaller percentage of the imaging field-of-view, a different geometry with the long snout, and a wide variety of head/neck sizes. While the fill factor for large breeds is similar to humans, for smaller dogs it would be approximately 50%. Furthermore, the majority of the dogs in the current study were mesaticephalic breeds, which tended to have a worse fill factor than the few included brachycephalic breeds. The development of veterinary-specific RF coils, particularly for mesaticephalic or dolichocephalic conformations, would increase the sensitivity by approximately a factor of two, with sensitivity approximately proportional to the inverse of coil diameter. Despite the hardware limitations, some of our best images shown in Figure 4 of a brachycephalic cadaver show what is potentially possible.

In conclusion, a brain scanning protocol consisting of T1w and T2w TSE sequences was successfully developed for a proprietary 0.05 T LF MRI by scanning a diverse population of canine cadavers. Comparative evaluation with a 1.5 T HF MRI showed that, despite the inherent loss of SNR and spatial resolution, the LF device has similar performance in the identification of a few anatomic features, within the scope of a non-inferiority margin of 15%. Notably, non-inferior identification of the LV and MA in T2w sequences, with inconclusive non-inferiority for the LV in the T1w sequences, is promising for the device's functionality for pediatric hydrocephalus neuroimaging. Furthermore, the overall higher mean P(LF+|HF+) in T2w sequences of 85.5% (SD 8.22%) for all 15 included variables suggests that the LF system may be able to detect more features reliably, provided that there is consistent image quality and improved study design. To overcome the post-mortem limitations and determine the full extent of the LF system's capabilities, further *in vivo* investigation is warranted, though this will present other technical challenges, such as compensating for temperature drift or minimizing electromagnetic interference, the severity of which may only become apparent *in vivo*. (O'Reilly et al. 2021) Future studies with larger sample sizes, non-degraded tissues, improved imaging criteria, and implementation of additional statistical analyses, such as a stricter non-inferiority margin or visual grading characteristic analysis, should provide a clearer assessment of the device's true clinical applicability.

## Supplementary Material

Supplementary material Supplementary Material

SupplementaryTables_ElisabethBurgers.xlsx SupplementaryTables_ElisabethBurgers.xlsx

## Data Availability

The low-field data supporting the findings of this study are openly available in Zenodo at https://doi.org/10.5281/zenodo.18873835, reference number 18873835.
