# Supplementary material for: Evaluation of a 0.05 T low-field MRI system for canine brain imaging via comparison with images acquired at 1.5 T field strength: a pilot cadaver study
Source: Vet Q. 2026 Sep 12;46(1):2720640. doi: 10.1080/01652176.2026.2720640 (PMC13573525; doi:10.1080/01652176.2026.2720640)
Supplement: Supplementary file 1 [file TVEQ_A_2720640_SM6918.docx]

**Supplementary Materials**

**Supplementary Table 1** Available cadaver signalment and clinical history. M=mesaticephalic, B=brachycephalic, FI=female intact, FS=female spayed, MI=male intact, MN=male neutered.

| **Cadaver** | **Reason for Euthanasia** | **Storage** | **Weight (kg)** | **Skull shape** | **Sex** | **Breed** |
| --- | --- | --- | --- | --- | --- | --- |
| 1 | Unknown | Frozen |  | M | Unknown | Unknown |
| 2 | Unknown | Frozen |  | B | Unknown | Unknown |
| 3 | Unknown | Frozen |  | M | FS | Mixed breed dog (small) |
| 4 | Open pyometra, owner declined treatment | Frozen |  | M | FI | Jack Russel Terrier |
| 5 | Unknown | Frozen |  | B | FS | Mixed breed dog (small) |
| 6 | Septic abdomen secondary to pyometra, owner declined treatment | Frozen |  | M | FI | Nova Scotia Duck Tolling Retriever |
| 7 | Unknown | Frozen |  | M | MI | Dachshund |
| 8 | Cardiopulmonary arrest under general anesthesia | Frozen |  | M | MI | Chihuahua |
| 9 | Quality of life, acute glaucoma | Frozen |  | M | MI | Mixed breed dog (small) |
| 10 | Unknown | Frozen |  | M | MI | Spanish Waterdog |
| 11 | Unknown | Frozen |  | M | FI | Miniature schnauzer |
| 12 | Cardiopulmonary arrest, post-operative hypophysectomy | Frozen | 5.6 | B | FS | Mixed breed dog (small) |
| 13 | Unknown | Frozen |  | B | MI | Mixed breed dog (small) |
| 14 | Unknown, dead on arrival at clinic | Frozen |  | M | FI | English Staffordshire Terrier |
| 15 | Unknown, dead on arrival at clinic | Frozen |  | M | MN | Shetland sheepdog |
| 16 | Unknown | Frozen |  | M | MN | Papillon |
| 17 | Quality of life, dysphagia | Fresh | 20 | M | FS | Laekense Belgian Shepherd |
| 18 | Unknown | Frozen | 22.8 | B | MI | Staffordshire Mix |
| 19 | Unknown | Frozen |  | M | MI | German wirehaired pointer |
| 20 | Unknown | Frozen |  | B | FI | French Bulldog |
| 21 | Unknown | Frozen |  | M | FS | Mixed breed dog (Belgian Malinois x Whippet) |
| 22 | Unknown | Frozen | 8.2 | M | FS | West Highland White Terrier |
| 23 | Unknown | Frozen |  | M | FI | Dutch Smoushond |

**Supplementary Table 2** Acquisition parameters for each sequence per cadaver.

See attached excel document.

**Supplementary Table 3** Reliability results per system for T1w sequences reported as Gwet's AC1 (95% CI).

See attached excel document.

**Supplementary Table 4** Reliability results per system for T2w sequences reported as Gwet's AC1 (95% CI).

See attached excel document.
